# Ovarian Cancer Immunotherapy and Personalized Medicine

**DOI:** 10.3390/ijms22126532

**Published:** 2021-06-18

**Authors:** Susan Morand, Monika Devanaboyina, Hannah Staats, Laura Stanbery, John Nemunaitis

**Affiliations:** 1 Department of Medicine, University of Toledo, Toledo, OH 43614, USA; susan.morand@rockets.utoledo.edu (S.M.); monika.devanaboyina@rockets.utoledo.edu (M.D.); hannah.staats@rockets.utoledo.edu (H.S.); 2 Gradalis, Inc., Carrollton, TX 75006, USA; lstanbery@gradalisinc.com

**Keywords:** ovarian cancer, immunotherapy, biomarker

## Abstract

Ovarian cancer response to immunotherapy is limited; however, the evaluation of sensitive/resistant target treatment subpopulations based on stratification by tumor biomarkers may improve the predictiveness of response to immunotherapy. These markers include tumor mutation burden, PD-L1, tumor-infiltrating lymphocytes, homologous recombination deficiency, and neoantigen intratumoral heterogeneity. Future directions in the treatment of ovarian cancer include the utilization of these biomarkers to select ideal candidates. This paper reviews the role of immunotherapy in ovarian cancer as well as novel therapeutics and study designs involving tumor biomarkers that increase the likelihood of success with immunotherapy in ovarian cancer.

## 1. Introduction

In the United States, 22,000 patients are diagnosed with ovarian cancer annually, making it the eleventh most common cancer among female patients and the fifth leading cause of cancer-related death in women [1,2]. Current front-line standard of care includes debulking surgery with platinum–taxane maintenance chemotherapy. Following front-line therapy, cancer will recur in 60–70% of patients with optimal debulking (<1 cm residual disease) and 80–85% of patients with suboptimal debulking (>1 cm residual disease), making the five-year survival reach about 45% [3,4,5,6]. Developments in front-line maintenance therapy have tried to extend this interval. Approved maintenance therapy involving bevacizumab or PARP inhibitors has shown efficacy to prolong progression-free survival (PFS) but not overall survival (OS), indicating that more effective maintenance therapy is needed [7,8]. Currently, most clinical trials focus on targeted approaches including more recent attempts at introducing immune therapeutics to the ovarian cancer treatment landscape.

Immunotherapy enhances the anticancer immune response through multiple approaches including but not limited to immunostimulatory cytokines, tumor antigen vaccines, and monoclonal antibodies targeting immunosuppressive ligands expressed by tumor cells (Table 1). The latter approach is principally aimed at immune checkpoint inhibition (ICI). Immune checkpoints include cytotoxic T-lymphocyte associated protein 4 and its ligand (CTLA-4:B7/CD80) and programmed death receptor-1 and its ligand (PD-1:PD-L1), which serve to distinguish pathogens from self-cells. When a T-lymphocyte encounters a peripheral cell, it seeks epitopes that match its T-cell receptor (TCR) affinity and determines if a pathogen or self-cell was encountered. In the presence of immune checkpoints such as PD-L1, T-cells sense that the epitope indicates a self-cell. In the absence of immune checkpoints, the T-cell identifies the target as pathogenic, and the killing response ensues [9]. Cancer cells upregulate immune checkpoints, thereby decreasing the local immune response and permitting immune evasion [9,10]. By binding CTLA-4, PD-1, or PD-L1, ICIs prevent the immune checkpoint interaction between the tumor and T-cell, thereby restoring T-cell cytotoxicity [11,12].

## 2. Molecular Profiling

Despite the ability of ICIs to produce durable responses in some patients, there remains a subset of patients who do not respond, including patients with tumors that exhibit PD-L1 expression. Thus, indication for immunotherapy is increasingly guided by molecular profiling that demonstrates immunogenic phenotypes, and new information is burgeoning in this field. Immunophenotype markers include tumor mutational burden (TMB), PD-1, and PD-L1 [13,14]. Other markers include homologous repair deficient and proficient (HRD, HRP) phenotypes and factors controlling the tumor microenvironment (TME) including the makeup of tumor-infiltrating lymphocytes (TILs) [15,16,17]. Together, these factors paint a picture of the immunogenicity of the cancer cell itself, the ability of the immune system to access the tumor, and the ability of immune cells to enact killing functions.

### 2.1. TMB

Tumor mutational burden describes the number of nonsynonymous mutations within a tumor sample and represents the degree of genomic instability as well as the likelihood of neoepitope appearance on the cell surface [13,18]. Neoepitopes are proteins unique to the cancer cell that are expressed on the cell exterior and are therefore accessible to the immune system. TMB thresholds have been defined differently by various research groups; in general, TMB is divided into high and low categories with TMB-high defined as >10 mutations/Megabase of DNA [19]. A TMB-high phenotype implies a large degree of mutated proteins, which may be expressed upon the cell surface as neoepitopes. It is well-documented that the TMB-high phenotype predicts a response to therapy with ICIs in solid tumors, which has been investigated in both preclinical and clinical studies of efficacy [19,20,21,22,23,24,25,26]. Recently, pembrolizumab, a monoclonal antibody targeting PD-1, was approved for use in any TMB-high (≥10) tumor, regardless of histology [27,28]. However, the positive relationship has not been consistently upheld in studies of ovarian cancers, where TMB has not been found to predict response to immunotherapy [19]. Furthermore, ovarian cancer is considered to be a “cold tumor” with TMB-low phenotype [29,30]. Median TMB in ovarian cancer is 3.6 mutations/Mb and mean TMB is 5.3 mutations/Mb, despite the expectation of a high TMB in the context of deficient DNA repair [31].

In general, TMB’s predictive ability is refined by considerations of other markers that may improve a patient’s likelihood of response to immunotherapy (e.g., combined TMB-high and PD-L1-high) or predict resistance to immunotherapy despite the TMB-high phenotype (e.g., combined TMB-high and high neoantigen intratumoral heterogeneity (ITH) [19,21,32,33,34,35]. It is estimated that 45% of ovarian tumors have high expression of PD-1/PD-L1, which is defined as greater than 10% of tumor cells in a tissue sample displaying greater than 10% surface expression of PD-L1 [36,37]. High expression of PD-1/PD-L1 is immunoinhibitory, and ICIs were designed to block this checkpoint-mediated immunosuppression. The high expression of PD-1/PD-L1 predicts response to ICIs independent of TMB status [12,23,29,38,39,40]. However, when combined, the two markers predict superior response than either marker independently [21,35,41]. To date, single biomarkers have been used as an indication for treatment; considerations of combined biomarkers may improve the precision of selecting patients most likely to respond to immunotherapy, especially in ovarian cancer.

### 2.2. HRD

Homologous repair deficiency (HRD) describes tumors with impaired response to DNA damage and is a biomarker predictive of response to poly-(ADP ribose) polymerase (PARP) inhibitors and platinum chemotherapy [15,42,43]. It is estimated that half (41–50%) of epithelial ovarian cancers are HRD, and over one in four ovarian cancer patients harbor germline mutations in HRD genes [44,45,46,47,48]. Somatic or germline mutations in *BRCA* genes are present in 25.7% of patients presenting with ovarian cancer, and *BRCA* mutant cells are classified as HRD [45,49]. HRD is associated with familial cancer genes including germline *BRCA* mutations in breast/ovarian cancer and mismatch repair (MMR) in Lynch syndrome. Interestingly, family history of cancer is positively associated with objective response rate (ORR), disease control rate (DCR), median time to treatment failure, and median OS following administration of ICIs, raising the question of whether HRD may be the mediator of this response to ICIs in familial cancers [50]. Other gene signatures are also indicative of an inability to repair DNA, including deficient MMR and other genes involved in the homologous recombination pathway including *RAD51*, *BARD1*, and *TP53* [15]. Tumors with MMR have shown great response to pembrolizumab, which is a PD-L1 inhibitor [51]. Consequently, pembrolizumab was approved as first-line treatment for MMR-deficient colorectal tumors [52,53,54,55]. However, *BRCA1/2*-mutant tumors have failed to demonstrate response to avelumab among other immunotherapies [56]. In this population, immune checkpoint inhibitors have experienced lower-than-expected rates of success [57,58,59].

Consequences of HRD include increased tumorigenesis, increased tumor mutations, and subsequently increased expression of tumor neoantigens [60]. HRD is associated with increased immunophenotype markers including TMB-high, increased CD3+ and CD8+ TILs, and increased levels of PD-1/PD-L1 compared to HRP tumors [51,61,62]. Despite the high prevalence of HRD in ovarian cancer, TMB is lower than expected [31]. A study of breast cancer samples found that HRD tumors showed increased PD-L1 expression in HRD tumors associated with activation of the stimulator of interferon genes (STING) pathway. The same studied identified increased CXCL10 and CCL5 expression 3.5- to 11.9-fold more than HRP tumors, with increased recruitment of peripheral blood mononuclear cells (PBMCs) mechanistically linked to CXCL10 and CCL5 expression rather than neoantigen expression. [63]. Dunphy et al. further elaborated on the non-canonical, antigen-independent recruitment of NK cells, M1-macrophages, T- and B-cells to the ovarian TME via CXCL10 and CCL5. The same group observed the recruitment of regulatory T-cells (T_regs_) and protumor M2-macrophages via the ATM-TRAF6-mediated “alternate STING pathway” producing IL-6 and TGF-beta. This ATM-TRAF6 also mediates the further upregulation of PD-L1, which partly explains the dearth of immune activation in ovarian cancer [15,64]. Thus, the STING pathway and alternative-STING pathway mediate immunomodulation in HRD tumors and depending on the molecular profile of the tumor can either activate or inhibit the immune response.

Zhang et al. recently reviewed trials of ICIs in DNA repair-deficient patients [65]. While the authors are not aware of any study examining a complete panel of HRD patients with ICIs, several groups have studied patient populations with a mutation in a select HRD gene/gene set, including MMR, *BRCA1/2*, *POLD1* or *POLE*, *MUTYH*, and *ERCC1*. Studies in the MMR and *BRCA1/2* populations include ovarian cancer patients, and the *POLD1/POLE* and *MUTYH* groups included other gynecologic cancers (endometrial). These studies are ongoing, with the exception of the aforementioned KEYNOTE-177 study, which demonstrated the efficacy of anti-PD-1 inhibitor therapy in the MMR-deficient population of colorectal cancer patients and conflicting results in the JAVELIN study of anti-PD-L1 therapy in non-small lung cancer, as well as ovarian cancer with *BRCA1/2* mutations [65,66,67,68].

### 2.3. Neoantigen Intratumoral Heterogeneity

Neoantigen ITH represents a variation between the neoepitopes expressed by cells within the tumor. High neoantigen ITH indicates that the cells within a tumor sample are non-uniform and carry distinct neoepitope profiles. By contrast, low neoantigen ITH indicates a relative uniformity of tumor cells, with similar neoepitope profiles expressed by most cells in the sample [33]. When neoantigen ITH is high, subclonal populations of cells persist, while the primary clonal population is targeted by the immune system. Subclonal populations are not targeted effectively within the tumor due to the decreased quantity of T-cells that are able to recognize the subclonal neoantigen [14]. McGranahan et al. found that high clonal neoantigen burden combined with low neoantigen intratumoral heterogeneity portended a good prognosis in patients with NSCLC and melanoma receiving anti-PD-1 or anti-CTLA-4 antibodies [33,69]. Neoantigen ITH is associated with different markers in the TME. Tumors with a high degree of clonal neoantigens (low neoantigen ITH) demonstrated significantly different expression of 25 genes, with IL-6 and PD-L1 as the most significantly upregulated proteins in the high neoantigen burden, low neoantigen ITH group. Other upregulated immunostimulatory genes included those involved with antigen presentation, immune cell migration, and effector T-cell function. Upregulated negative immunomodulatory genes included *PD-1* and *LAG-3* [33]. Thus, high neoantigen burden combined with homogeneity, or low neoantigen ITH, is associated with an inflamed TME as well as modulation of the immune system, indicating an association between immunoediting and ITH score.

### 2.4. Tumor Infiltrating Lymphocytes

Another biomarker that may predict response is the quantification of tumor infiltrating lymphocytes (TIL) which determines immune cell infiltration of the tumor microenvironment (TME) and predicts good response to immunotherapy [17]. Attention has been drawn to the role that T-cells, NK cells, and more recently B cells play in the TME [16]. Analysis has been complicated by somewhat contradictory findings relating TILs to survival and prognosis. For example, a study of the TME in melanoma found that increased numbers of TILs corresponded with tumor aggressiveness and stage. This same study found that high levels of CD69+, a marker of lymphocyte activation, correlated with survival, indicating that considerations of quantity and quality of TILs are important for predicting prognosis [70]. In ovarian cancer, the presence of TILs corresponds with an increased PFS and OS [71,72,73]. Regardless of tumor stage, the presence of TILs in ovarian cancer portends a good prognosis, particularly when CD8+ T-cells are present [72,74]. Westergaard et al. noted that the TIL profile of ovarian tumors is similar to that of melanoma but with a higher proportion of CD4+ T-cells compared to CD8+ T-cells. These T-cells commonly displayed the CD45RO^+^CCR7^−^CD62L phenotype, which is consistent with effector memory T-cells [75,76]. Over half of the patients analyzed demonstrated T-cell recognition of autologous tumor cells, and antigen-specific TILs were isolated and expanded. A second difference between ovarian cancer tissue and melanoma was that the ovarian TILs were less frequently tumor-reactive, which may be explained by elevated TIL expression of LAG-3, which is a marker of T-cell exhaustion. Westergaard et al. attributed the high expression of LAG-3 to elevated levels of IL-2 in the TME [75].

Sakellariou-Thompson et al. similarly studied the TME in ovarian cancer patients. They found a higher proportion of CD3+ T-cells in metastatic ovarian cancer samples than in tissue derived from primary tumors but similar TME profiles in samples from pre-treated chemotherapy patients versus chemo-naïve patients [77]. In contrast to Westergaard et al., Sakellariou-Thompson’s group found a higher proportion of CD8+ T-cells to CD4+ T-cells, with a ratio of 1.5. This is consistent with prior studies of melanoma TME by the same group, indicating that ovarian cancer has similar infiltration by the immune system [77,78].

Autologous vaccines are focused on activating “neo-antigen” T-cells to target tumor mutations specific to each patient [79,80,81]. Previously, autologous TILs therapy has been shown to be effective in melanoma [82,83]. In ovarian cancer, TILs were shown to correlate with improved clinical outcome [77,84,85]. CD8+ TILs found within the epithelium express integrin CD103 on their cell membrane, which can bind to E-cadherin—a known tumor suppressor protein mediating epithelial adhesion [86,87]. In a clinical study, seven patients with advanced epithelial ovarian cancer (EOC) were treated with a single dose of autologous TILs expanded with IL-2 where five out of seven (71.4%) exhibited a complete or partial response, 1 and 4 respectively. In the group with combination therapy of two to three doses of TILs and chemotherapy of cisplatin, seven out 10 achieved a complete response and two out of 10 achieved a partial response, meaning that the total response rate was nine out of 10 (90%) [86]. These results illustrate the potential benefit of TILs against cancer through enhancing the immune system.

TIL markers corresponding with survival include the expression of CD8+, CD4+, granzyme B, MHC-I on tumor cells, and MHC-II on intratumoral antigen-presenting cells [17,88,89,90,91,92]. By contrast, the high expression of M2-macrophages and FOXP3+ regulatory T-cells (T_regs_) correlates with poor survival [17,93,94,95,96,97]. T_regs_ secrete TGF-beta, an immunosuppressive molecule, and they are associated with the decreased activation of T-cells and decreased endogenous tumor-associated antigen (TAA) specificity. They have an inversed correlation with survival in ovarian cancer [95]. The role of CD20+ B cells has been equivocal, with early studies suggesting a negative prognostic role and newer studies demonstrating a correlation with improved survival [93,98].

Recent studies have identified tertiary lymphoid structures (TLS) in the tumor microenvironment [99]. These structures are an ectopic aggregation of lymphoid tissues containing B-cells, T-cells, and dendritic cells, and they are associated with improved survival [100,101]. Ghisoni et al. reviewed common TME markers in ovarian cancer including T_regs_, tumor-associated macrophages (TAMs), myeloid-derived suppressor cells (MDSC), exosomes, adipocytes, and cancer-activated fibroblasts (CAFs). Per the authors, each of these markers can impair the activity of TILs [102]. Beyond guiding immunotherapy, TILs have a role in directing surgical considerations. In patients with high expression of CD8+ T-cells, patients maintain similar outcomes regardless of debulking surgery success; in contrast, patients with low levels of CD8+ T-cells have superior outcomes with optimal debulking surgery compared to suboptimal debulking [103].

TMB-high, HRD, ITH-low, high TIL with immunogenic phenotype, and PD-L1 expression are among the biomarkers hypothesized to predict response to ICIs. In ovarian cancer, the lower-than-expected response to ICIs may be improved with focused selection of patients based on molecular profiling. A combination of several biomarkers may be needed to predict response. The clinical utility of these biomarkers has yet to undergo vigorous testing, although many trials are underway.

## 3. Immune Therapy Clinical Trials in Ovarian Cancer

Despite ovarian cancer’s high proportion of HRD tumors with suspected high TMB, increased infiltration by CD8+ TILs, and high expression of tumor antigens capable of eliciting spontaneous anti-tumor responses, initial attempts at immunotherapy in ovarian cancer were largely underwhelming [47,48,73,104]. The JAVELIN Ovarian 100 trial involved avelumab, a PD-L1 inhibitor, as maintenance therapy in stage III/IV previously untreated epithelial ovarian cancer. Arms included chemotherapy followed by avelumab; avelumab plus chemotherapy followed by avelumab; and chemotherapy followed by observation as control. The study was discontinued prior to completion according to predetermined futility boundaries and failure to demonstrate improvement in PFS compared to control (11.1 months, 11.0 months, and 10.2 months, respectively across arms) [105]. Similarly, IMagyn050/GOG3015/ENGOT-OV39 evaluated atezolizumab, a PD-L1 inhibitor, compared to placebo combined with paclitaxel, carboplatin, and bevacizumab in patients with advanced epithelial ovarian cancer. Researchers found no significant difference in median PFS in the intent-to-treat population compared to placebo (19.5 months vs. 18.5 months, HR 0.92) nor the PD-L1 positive population compared to placebo (20.8 months vs. 18.4 months, HR 0.80) [106,107]. Negative outcomes in both of these studies indicate checkpoint inhibitor use in ovarian cancer may require additional biomarker efficacy analysis to determine a potentially sensitive population.

Monotherapy of pembrolizumab was studied more extensively through the KEYNOTE-158 trial in patients with cytologically confirmed noncolorectal high microsatellite instability (MSI-H) and mismatch repair-deficient (dMMR) solid tumors including ovarian, endometrial, and gastric cancer that were previously treated with standard chemotherapy. The patients were considered to have a high tumor mutational burden as at least one out of four tumor mismatch repair proteins, MLH1, MSH2, MSH6, and PMS2, was missing. In addition, two out of the five allelic loci shift of BAT25, BAT26, Di 5S346, Di 2S123, or Di 17S250 was determined as high microsatellite instability. Pembrolizumab was given at 200 mg once every three weeks for two years or until disease progression. For the 15 ovarian cancer patients previously resistant to treatment, an objective response was noted in five patients (33.3%), and a complete response was noted in three patients. As the study is still under progress, further results are awaited. A total of 223 patients were enrolled in the study, and 23 (9.9%) reported a complete response with 57 (24.5%) reporting a partial response. The ORR was stated as 34.3% (95% CI, 28.3–40.8). The positive outcomes in this study supports the FDA approval in May 2017 for pembrolizumab use in metastatic MSI-H/dMMR solid tumors including ovarian cancer [52]. The overall results of the study including other types of tumor are also of importance.

Pembrolizumab has also been investigated in recurrent ovarian cancer in the KEYNOTE-100 trial. In this trial, the first 100 patients enrolled were used to determine the combine positive (CPS) PD-L1 cut-off score. From this set of patients, CPS scores of 1 and 10 were used for efficacy analysis [108]. Two cohorts of recurrent ovarian cancer patients were enrolled; Cohort A consisted of patients who received one to three previous lines of therapy with a platinum-free interval of 2 to 12 months. Cohort B patients received four to six prior lines of therapy and a platinum-free interval of ≥3 months. Correlation of response with higher CPS score was demonstrated in both cohorts, and responses ≥6 months were observed. However, antitumor activity was described as modest [108,109].

The MIMOSA study was a phase III, double-blind, placebo-controlled, multicenter trial that assessed whether abagovomab maintenance therapy prolonged recurrence free survival (RFS) and OS in ovarian cancer patients in first clinical remission. Abagovomab is a murine monoclonal antibody that targets the tumor-associated antigen CA-125. The study included stage III or IV ovarian cancer patients in complete clinical remission after primary surgery and platinum- and taxane-based chemotherapy. Abagovomab or placebo was administered once every two weeks for six weeks and then once every four weeks until recurrence or up to 21 months after random assignment of the last patient. Of the 888 patients included, 81.5% had the serous papillary subtype, 85.9% were stage III, and 80.9% had a cancer antigen 125 ≤ 35 U/mL after third cycle. Mean exposure to study treatment was 449.7 days. No benefit in RFS was demonstrated when stratified by tumor size (≤1 cm, >1 cm) (HR 1.099 95% CI, 0.919–1.315; *p* = 0.301). Similarly, no benefit in OS was demonstrated (1.150 95% CI, 0.872 to 1.518; *p* = 0.322). The OS rate at two years was 80% in both groups, with SE equal to 1.71 and 2.43 for abagovomab and placebo groups, respectively. By the final visit, the median anti–anti-idiotypic antibody level was 493,000.0 ng/mL, indicating that a robust immune response was obtained. The trial concluded that while treatment was safe and measurable immune response was obtained, maintenance therapy with abagovomab in first remission does not prolong RFS or OS. The study was ultimately terminated due to a failure to meet the primary end point (RFS) [110].

Farletuzumab use in combination with carboplatin and taxane in ovarian cancer patients during first platinum-sensitive relapse was assessed in a randomized, double-blind, placebo-controlled, phase III study. Farletuzumab is a humanized monoclonal antibody that targets folate receptor-α (FRα). FRα is expressed in 80–100% of epithelial ovarian cancers (including primary peritoneal and fallopian tube cancers) and is absent from normal tissue and could indicate a negative prognostic factor with chemotherapy [111,112,113,114]. The primary end point of the trial was PFS; there was also a subgroup analysis done by baseline CA-125 and farletuzumab exposure levels. PFS was 9.0, 9.5, and 9.7 months for the placebo, farletuzumab 1.25 mg/kg, and farletuzumab 2.5 mg/kg groups, respectively and was not statistically different from the placebo group (HR 0.99 (95% CI, 0.81 to 1.21) and 0.86 (95% CI, 0.70 to 1.06) for the farletuzumab 1.25 mg/kg and 2.5 mg/kg groups versus placebo, respectively). In the subgroup analysis, baseline CA-125 levels not more than three times the upper limit of normal correlated with prolonged PFS (median, 13.6 vs. 8.8 months; HR 0.49; *p* = 0.0028) and OS (HR 0.44; *p* = 0.0108) for farletuzumab 2.5 mg/kg versus placebo. Subgroup analysis of farletuzumab exposure above the median, regardless of dose, showed significantly better PFS versus placebo. In addition, patients with lower CA-125 levels at baseline did show improvement on the higher dosage (2.5 mg/kg) of farletuzumab in both PFS and OS. Adverse events were similar between groups, and treatment was tolerated. The trial concluded that neither dosage of farletuzumab significantly prolonged PFS, but there may be unidentified subgroups that could benefit from farletuzumab therapy [114,115,116,117].

Anti-FRα T-cells can be generated through the modification of autologous T-cells ex vivo in order to respond to FRα+ tumor cells with the addition of IL-2 [118,119]. Dual-specific T-cells can also be generated from T-cells with endogenous specificity for allogenic antigen along with anti-FRα activity without the need for IL-2 [118]. Fourteen patients with FR+ metastatic EOC after standard therapy were enrolled in a phase I clinical trial and treated with one to three cyclical doses of anti-FRα T-cells alone or dual-specific T-cells. Although tumor responses were lacking in computerized tomography scans and serum CA-125 levels, the lack of response could be due to the route of intravenous delivery, which could have been improved with intraperitoneal delivery with enhanced T-cell trafficking to tumor site. In addition, the responsiveness of T-cells to FR antigen was noted, but the expansion of T-cells in patients could not be determined. The study stated that the treatment was well tolerated in patients, with five out of 14 patients exhibiting grade 3 or 4 adverse events. As the doses of the treatment were considered to be low and safety was demonstrated, it was concluded that future studies could increase the dose of the T-cell treatment [118]. A more recent phase I clinical trial also studied the treatment of FRα peptide vaccine with six monthly doses alongside cyclophosphamide to augment antigen-specific immune responses in 22 ovarian or breast cancer patients. This study reported a significant elevation of interferon-gamma (IFN-γ) T-cell frequency in patients after treatment (*p* = 0.00003) and corresponded with a clinical benefit. All 22 patients survived at the 2-year follow-up. The median RFS time in ovarian cancer patients was 528 days, demonstrating that the peptide vaccine could augment the anti-tumor response. The FRα peptide vaccine was given to all patients regardless of FRα expression, meaning that individuals who are determined to have FRα expression could have greater response rates [120].

Earlier attempts at combination therapy of immune and chemotherapy have also been attempted using motolimod, Toll-like receptor 8 (TLR8) agonist and pegylated liposomal doxorubicin (PLD). In a phase II study at 105 study centers with 297 total ovarian cancer patients with persistent disease following primary chemotherapy, the results reported that the combination of motolimod and PLD did not significantly improve OS, which was 18.1 months compared to 18.9 months for the placebo (HR, 1.22; *p* = 0.923). The PFS (HR 1.21, *p* = 0.943) and ORR were also not affected with the addition of motolimod. Motolimod and PLD were well tolerated, and the rate of treatment-emergent adverse events occurred at a similar rate of incidence between both study groups. Although the innate immune response was activated in patients treated with motolimod as seen with the increase of cytokines, IL-1, IL-6, and TNFα, in plasma, the immune response did not correlate with significant clinical outcomes. Furthermore, a subset analysis of motolimod-treated patients with increased baseline IFN-γ, TNF-α, or IL-12p4 did show a significant improvement in OS compared to patients without increased baseline cytokines. These results support the hypothesis that the combination immunotherapy was advantageous in patients with a prior robust immune function [121].

The VITAL trial was a double-blind, placebo-controlled, phase IIb trial that assessed gemogenovatucel-T (Vigil) as maintenance therapy in stage III/IV ovarian cancer. Vigil is an autologous tumor cell vaccine manufactured from harvested tumor tissue and transfected ex vivo with a multigenic plasmid encoding the human granulocyte macrophage colony-stimulating factor (GMCSF) gene and a bifunctional short-hairpin RNA (bi-shRNA) construct, which reduces the expression of furin and downstream TGF-β1 and TGF-β2 [122]. TGF-β is highly expressed in malignant ovarian tissue compared with non-malignant tissue and is correlated with poor prognosis. Overexpression is associated with tumor cell proliferation and metastasis and is increased in patients with suboptimally debulked ovarian cancer [123,124,125]. Phase I trial results demonstrated safety in advanced solid tumors and identified a survival advantage in γ-IFN-ELISPOT positive patients [126,127,128,129]. Additionally, Vigil was shown to increase CD3+/CD8+ circulating T-cells [130]. Subsequently a phase IIb trial of women with stage III/IV high grade serous, endometroid, or clear cell ovarian cancer who were in complete response to front-line treatment was conducted. RFS was improved in patients receiving Vigil versus placebo; however, the improvement was not statistically significant (11.5 vs. 8.4 months HR 0.69 CI 0.44–1.07; *p* = 0.078). Preplanned subgroup analysis revealed a statistically significant advantage in *BRCA1/2* wild-type patients who received Vigil versus placebo (HR 0.51 CI 0.30–0.88; *p* = 0.02) from randomization. Moreover, OS appeared improved in the *BRCA1/2* wild-type Vigil treated patients compared to placebo (not reached vs. 41.4 months respectively; HR 0.49 90% CI 0.24–1.01; *p* = 0.049) [131]. Additionally, Vigil demonstrated efficacy in the homologous recombination population. RFS in the HRP Vigil-treated patient population improved to 10.6 months vs. 5.7 months in placebo-treated patients (HR 0.386 90% CI 0.199–0.750; *p* = 0.007), and OS was not reached in Vigil-treated vs. 26.9 in placebo-treated patients (HR = 0.342 90% CI 0.141–0.832 *p* = 0.019) [132]. Vigil is the first immunotherapy to show efficacy in the *BRCA* wild-type and HRP population. The mechanism for Vigil efficacy in this population is likely due to the ability of Vigil to educate T-cells to the relevant clonal neoantigens, which are highly represented in tumors that retain the ability for DNA repair.

## 4. Ongoing Clinical Trials

While previous trials have not been successful at showing efficacy with the inclusion of immunotherapies in ovarian cancer treatments, there is still ongoing investigation to uncover biomarkers to predict response to immune therapeutics in ovarian cancer patient subgroups. Additionally, combination studies of immune therapeutics with other therapies are underway. There are many trials currently in progress studying the effects of various immunotherapies in ovarian cancer, and here, we will review some trials in progress that utilize biomarkers to stratify patient response, which are summarized in Table 2.

## 5. Current Directions within Personalized Immunotherapy

Inducing an anti-tumor T-cell response with the TME is important for immunotherapy to be effective. There are many approaches currently being investigated to achieve this goal including dendric cells, autologous tumor vaccines, and other combination therapies.

### 5.1. Dendritic Cells

One approach includes pulsating autologous dendritic cells (DCs) with a tumor peptide [142,143]. The selected peptide, Wilms’ tumor protein 1 (WT1), is normally expressed in kidneys, Sertoli cells of the testis, and granulosa cells of the ovary. However, the frequency of WT1 expression in EOC tissues was noted to be at 78% in a sample of 100 patients, where WT1 was associated with tumors of higher grading (*p* = 0.006) and staging (*p* = 0.002) [144,145]. A phase I/II clinical study examined the role of the Wilms’ tumor protein 1 (WT1), which is an indicator for poor prognosis in ovarian cancer at a 5-year survival rate of 47%. Vaccination with autologous DCs induced a significant CD8+ T-cell response against WT1 in patients with ovarian, breast, and gastric cancer who presented with a WT1 mutation (*p* < 0.05). Two out of the 10 (20%) patients reported partial response, and seven out of the 10 (70%) patients presented with stable disease after treatment where three out of the seven had reported tumor shrinkage on CT scan. All adverse events were grade 1 or 2; therefore, treatment was determined to be safe and well tolerated [142]. This study demonstrates the need for personalized vaccinations to target specific mutations in patient subsets, which could produce improved outcomes.

### 5.2. Autologous Vaccines

Autologous vaccines could also be vital to the changing role of immunotherapy in cancer management [146]. One such combination is autologous vaccines in order to enhance immune responses to attack tumor cells and decrease tumor evasion simultaneously. One preliminary study analyzed combination therapy in six late-stage metastatic ovarian cancer patients resistant to chemotherapy. Ipilimumab, an anti-CTLA4 antibody, was followed by surgery and ex vivo expanded autologous TILs, IL-2, and nivolumab, an anti-PD-1 antibody. The study reported results of one patient with partial response and five with stable disease after 12 months, and the median progressive-free survival was determined to be 86 days with a range of 84 to 342 days. These results were also compared to previous results without ipilimumab treatment, where the success rate of ex vivo expanded autologous TILs increased with ipilimumab corresponding with an increased CD8+ T-cell activity [147]. Overall, the study highlights the beneficial effect of combination therapy of autologous vaccines with ICIs. Another autologous vaccine discussed previously, Vigil, which educates T-cells to the relevant clonal tumor neoantigens and increases peripheral circulating CD3+/CD8+ T-cells in combination with ICIs is a logical next step. A phase I trial investigated the combination of Vigil and atezolizumab in relapsed ovarian cancer patients. Investigators found that the timing of administration was important for not only efficacy but also safety. Administering Vigil before atezolizumab increased efficacy and decreased treatment-related adverse events associated with atezolizumab. OS was not reached in the Vigil first treatment arm and 10.8 months in the atezolizumab first arm (HR 0.33). Previous phase IIb trial results of Vigil revealed increased clinical benefit in *BRCA* wild-type patients, which was also suggested in this trial (NR in Vigil first vs. 5.2 in atezolizumab first HR 0.16, *p* = 0.027) [148]. The safety profile and clinical benefit observed in the small cohort of patients suggests that continued analysis is warranted.

### 5.3. Combination Therapeutic Approaches

Research studies evaluating the responses to combination therapy of autologous DC vaccine with ICIs and chemotherapy are also important to consider. One was a phase I study with recurrent ovarian cancer patients consisting of three treatment groups. The first group received monotherapy of the autologous DC vaccine pulsated with tumor cells, while the second received the autologous DC vaccine combined with bevacizumab, and the third received the vaccine and bevacizumab followed by cyclophosphamide. The results reported that the CD4+ and CD8+ T-cells increased significantly after vaccination. Out of the 25 patients treated, two were reported to have partial response, while 13 reported stable disease for a median of 14 months after vaccination. In addition, the results were analyzed further based on response to vaccine, which was determined through T-cell recognition of tumor cells. Among the 11 out of 25 that responded to the vaccine, the 2-year survival was 100%; however, the 2-year survival for non-responders was 25%. This study is critical in illustrating that the treatment could be vital in improving outcomes if delivered to the right patients with the greatest chance of response. As a result, determining factors for response to treatment is highly beneficial and should continue to be evaluated. Another important aspect of the study focuses on the benefit of combining cyclophosphamide with bevacizumab and the autologous DC vaccine. The group treated with cyclophosphamide in combination reported that eight out of 10 patients responded to the vaccine, whereas only three out of 12 responded without cyclophosphamide. Further serum analysis shows that an increase in TGF-β was reported after vaccination and again after cyclophosphamide administration, correlating to the responsiveness of the vaccine [149]. Continued research is warranted to illustrate the comprehensive effects of the autologous vaccines, which could provide additional benefit when supplemented with prior thoroughly researched and potent therapeutics to deliver personalized medicine.

These studies indicate positive results related to cancer vaccination. Additionally, a combination of vaccination and immunotherapy may be an attractive next step to sensitize tumors to the neoantigen repertoire. More research is needed to develop this strategy and also elucidate biomarkers to predict response.

T-cell proliferation, function, and recruitment are negatively regulated by tumor-associated macrophages (TAMs) [150,151,152,153]. Both preclinically and clinically, targeting TAMs has been successful and improved the benefit seen with traditional therapies: for example, chemotherapy and immunotherapy [153,154,155]. Therefore, targeting TAMs is an attractive therapeutic strategy and may work synergistically with immunotherapy. However, TAMs are also responsible for antigen presentation within the TME. Consequently, reprogramming of TAMs in order to take advantage of this function has also been explored. One approach is the use of CD40 antibody in combination with chemotherapy in a murine model of pancreatic cancer. This approach resulted in an increase of T-cells within the TME [156].

Autophagy can also play an important role in response to immunotherapies [157]. In ovarian cancer, elevated levels of MHC-II are associated with better prognosis and overall survival. Autophagy is one mechanism by which cells can increase the amount of neoantigens for the presentation by MHC-II. The regulation of autophagy is complex, and one pathway involves *BRCA1/2*. In *BRCA1/2* mutant cells, there is an increase in autophagy compared to *BRCA1/2* wild type [157]. Therefore, in *BRCA* wild-type tumors, autophagy inducers combined with immune therapies including checkpoint inhibitors and autologous tumor vaccines may be a logical approach. However, autophagy inhibitors could also be used to enhance or resensitize tumors to chemotherapy [158]. Autophagy is upregulated in response to stress, including chemotherapy. Preclinical studies have found that autophagy inhibitors can work synergistically with chemotherapy to decrease cell viability [159,160]. The use of autophagy inhibitors or inducers may be context dependent.

## 6. Conclusions

Initial studies indicated that ovarian cancer may be immunogenic due to several factors including homologous repair deficiency secondary to high rates of *BRCA* mutation. However, immunotherapies have had less success in ovarian cancer than in most other immunogenic tumor types such as NSCLC and melanoma. Strategies are being adapted to improve the efficacy of immunotherapy application to ovarian cancer, including selecting patients based on immune profiling, such as MSI-H/dMMR, HRD, and combining ICI with other therapeutics. Further research is necessary to fully characterize immune characteristics common to ovarian cancer, determine ideal markers for response, and refine the selection of patients eligible for therapy. Due to the complex immune landscape in ovarian cancer, more than one biomarker may be needed to accurately predict response. Combinatorial therapeutics appear an optimistic option for maximizing therapeutic benefit, and further analysis of efficacy and risk is necessary.

## Figures and Tables

**Table 1:** Types of cancer immunotherapies.

Type of Immunotherapy	Example
**Cancer Vaccines**	Provenge, Vigil
**Immune modulators**	Checkpoint inhibitors Immune regulatory cytokines
**Targeted antibodies**	Monoclonal antibodies
**Adoptive cell therapy**	CAR-T therapy in leukemia and lymphoma

**Table 2:** Clinical trials utilizing biomarker stratification analysis.

Trial Name	Short Description	Experiment Arms/Cohorts	Biomarker Stratification
KEYNOTE-158	Phase II, two arm, open-label trial investigating pembrolizumab and evaluating predictive biomarkers in subjects with advanced solid tumors	Arm 1: Pembrolizumab 200 mg Arm 2: Participants failed at least one line of therapy and have TMB high.	TMB high
NCT03428802 [133]	Phase II, single-arm, open-label trial studying the use of pembrolizumab in patients with metastatic, recurrent, or locally advanced solid tumors and genomic instability	Arm 1: Pembrolizumab and lab biomarker analysis	Response rate will be stratified by mutation type (*POLE* and *POLD1* versus *BRCA1/2*) Patient/clinical outcomes will be stratified by PD-L1 expression and presence of PD-1/PDL-1 polymorphisms and presence of immunoregulatory gene mutations (via deep sequencing) Response will be stratified by presence of immunogenic neoantigens (via exome sequencing) and expression of checkpoint genes, immune-regulatory modules, or non-coding RNAs including repetitive RNAs and retroelements (via RNA sequencing)
DUO-O [134]	Phase III, randomized, double-blind, placebo-controlled, multicenter trial studying the use of durvalumab with chemotherapy and bevacizumab followed by maintenance durvalumab, bevacizumab, and olaparib in advanced ovarian cancer	Arm 1: Platinum-based chemotherapy with bevacizumab and durvalumab placebo followed by maintenance bevacizumab, durvalumab placebo, and olaparib placebo Arm 2: Platinum-based chemotherapy with bevacizumab and durvalumab followed by maintenance bevacizumab, durvalumab, and olaparib placebo Arm 3: Platinum-based chemotherapy with bevacizumab and durvalumab followed by maintenance bevacizumab, durvalumab, and olaparib t*BRCA*m Cohort: Platinum-based chemotherapy with bevacizumab and durvalumab followed by maintenance bevacizumab, durvalumab, and olaparib (bevacizumab is optional)	Somatic *BRCA* mutation status
V3-OVA [135]	Phase II, single-arm, open-label trial studying the use of vaccine V3-OVA in ovarian cancer	Arm 1: V3-OVA vaccine (containing ovarian cancer antigens)	Secondary outcomes will assess the effect on level of serum tumor markers compared to baseline (including CA-125)
AdORN [136]	Phase I/II, single-arm, open-label trial studying the use of atezolizumab with neoadjuvant chemotherapy in interval cytoreductive surgery in patients with newly diagnosed advanced-stage epithelial ovarian cancer	Arm 1: Atezolizumab, carboplatin, and paclitaxel (and optional bevacizumab)	PFS will be stratified based on the expression of PD-L1, tumor-infiltrating lymphocytes, immune checkpoint receptors, and cytokines and gene expression profiles Each of those subsets will be further stratified by *BRCA* mutation status and tumor mutation profile
OLAPem [137]	Phase II, single-arm, open-label trial studying the use of olaparib monotherapy and olaparib and pembrolizumab combination therapy in ovarian cancer	Arm 1, Cohort 1: Olaparib before surgery Arm 1, Cohort 2: Olaparib and pembrolizumab before surgery	Therapeutic effect will be stratified by biomarkers (germline mutations), change in tumor-infiltrating lymphocytes, and tumor mutation burden
NCT02983799 [138]	Phase II, non-randomized, open-label trial studying the use of olaparib in patients with platinum-sensitive, relapsed, high-grade serous or high-grade endometrioid epithelial ovarian, fallopian tube, or primary peritoneal cancer that have different HRD tumor status and have received at least 1 prior line of chemotherapy	Arm 1: Germline *BRCA*m given olaparib Arm 2: Somatic *BRCA*m and germline *BRCA*wt given olaparib Arm 3: myChoice^®^ HRD positive and *BRCA*wt given olaparib Arm 4: myChoice^®^ HRD negative and *BRCA*wt given olaparib	Experimental arms stratified by HRD and *BRCA* mutation status Objective response rate will be stratified by HRD status as per HRRm gene panel assessment in *BRCA*wt cohorts 3 and 4
BOLD [139]	Phase II, single-arm, open label trial studying the use of bevacizumab, olaparib, and durvalumab in patients with relapsed advanced epithelial ovarian cancer	Arm 1: Bevacizumab, olaparib, and durvalumab combination	Response to treatment (evaluated by immune-related response criteria) will be stratified by tumor mutation burden, homologous repair status, and tumor immune infiltrate and immune check point status (PD-1/PDL-1 driven versus other immune check points involved).
AMBITION [140,141]	Phase II, randomized, multicenter, open label trial for HRD+ patients and a biomarker-driven multiple-arm phase II trial for HRD- patients studying the use of various combination therapies in the treatment of platinum-sensitive recurrent ovarian cancer	Arm 1: Olaparib plus cediranib Arm 2: Durvalumab plus olaparib Arm 3: Durvalumab plus chemotherapy (paclitaxel, topotecan, or pegylated liposomal doxorubicin) Arm 4: Durvalumab plus tremelimumab and chemotherapy (paclitaxel, topotecan, or pegylated liposomal doxorubicin) Arm 5: Durvalumab plus tremelimumab and paclitaxel	Patients HRD and PD-L1 status and presence of biomarkers will be evaluated and used to allocate treatment arms HRD+ patients will be randomly allocated to Arm 1 or 2 HRD- patients will be allocated to Arm 3 or 4 based on PD-L1 expression (allocation to Arm 3 if high PD-L1 expression and to Arm 4 if low PD-L expression)

These ongoing phase I/II/III trials have shown evidence of increased response in ovarian cancer patients treated with immunotherapy, often in combination with other therapies. Additional studies investigating biomarkers correlated with increased and decreased response are critical. There is great therapeutic potential in the use of immunotherapy in ovarian cancer, and there appears to be patients that do respond; however, more research needs to be done before we can understand its full potential.

## Data Availability

Not applicable.
